# Social/Emotional Health, Mental Health and Quality of Life among Adults with Comorbid Diabetes and Hypertension: A Population-based Cross-sectional Study

**DOI:** 10.13023/jah.0601.08

**Published:** 2024-09-01

**Authors:** Ranjita Misra, Sara Nayeem

**Affiliations:** West Virginia University; West Virginia University

**Keywords:** Appalachia, diabetes mellitus, hypertension, social/emotional factors, mental health, quality of life, rural

## Abstract

**Introduction:**

West Virginia has a disproportionately large population of rural adults with diabetes and hypertension, two common chronic, comorbid conditions that represent a national economic, social, and public health burden. Anxiety, depression, and severe mental illness are associated with poor motivation to engage in coping/self-care behaviors and related increased morbidity/mortality.

**Purpose:**

This study examines the relationship between self-reported mental health, selected social and emotional health factors, health-related quality of life (HRQoL), and clinical outcomes among adults with comorbid diabetes and hypertension.

**Methods:**

This cross-sectional study consisted of 75 participants who participated in a diabetes and hypertension self-management program (DHSMP) in West Virginia. Baseline measures (2018–2019) were used to explore associations and included demographics, self-rated mental health, diabetes distress, HRQoL, HbA1c, and blood pressure. One-way ANOVA was performed to compare mentally healthy v. unhealthy participants by their demographics, diabetes distress and its domains, HRQoL and its domains, and clinical outcomes.

**Results:**

The mean age and BMI were 60.8 ± 12.2 and 36.4 ± 8.1, respectively, indicating that the average participant was older and obese. Participants who self-reported fair or poor mental health had significantly higher BMI, higher diabetes distress, and lower HRQoL. Participants with good to excellent mental health had lower systolic blood pressure.

**Implications:**

Findings indicate the potential role of social and emotional health on clinical outcomes and HRQoL among patients with comorbid chronic conditions, especially for older obese patients. Future studies with larger sample sizes should explore tailoring lifestyle and educational programs to address these factors for improved health outcomes.

## INTRODUCTION

Diabetes and hypertension are two common chronic conditions in West Virginia (and the U.S. more widely), and mental health influences the individual course of these conditions. Approximately 16.0% of West Virginian adults have diabetes, and 41.0% have high blood pressure; for both, the state ranks first in prevalence nationally.^1^ Furthermore, three-quarters of adults with diabetes also have hypertension, and patients who have hypertension alone often show evidence of insulin resistance. Diabetes and hypertension have significant overlap in risk factors (e.g., ethnicity, lifestyle determinants, dyslipidemia, and complications).^2^ Mental health plays a key role in the self-management and outcome of patients living with two or more chronic conditions (defined as multimorbidity). While the interaction between physical and mental health is complex and multifactorial,^3^ there is growing evidence that individuals with long-term mental health challenges face higher risk of developing chronic conditions.^3^

It is important to assess the potential role of mental health in disease self-care and outcomes for individuals with diabetes and/or hypertension. Previous studies have shown that diabetes and hypertension are linked to depression and anxiety disorders, conditions associated with compromised mental health status.^3^ These conditions foster negative emotions in patients and increase their risk of developing both poor mental *and* physical health.^4^ For example, in a study of patients with chronic hypertension, negative emotions and stress increased the likelihood of medication non-adherence.^4^ A similar link exists between depression and regimen adherence among patients with diabetes.^5^ This suggests the need for clinicians to attend to emotional health as they treat patients.^4^

Adults with multimorbidity can also experience mental, physical, and economic difficulties related to diagnosis, self-care and treatment—these in turn can negatively affect their health-related quality of life (HRQoL) and its domains— physical, psychosocial, and social functioning.^6^ However, perceptions of the impact of multimorbidity on patients’ HRQoL differs between patients and their healthcare providers. For example, while patients considered physical functioning, diet, and emotional well-being as the three most important factors affecting HRQoL, providers did not consider emotional well-being as important, and ranked it considerably lower than that of patients.^7^

Physical and mental well-being, along with physical/social functioning, are affected by social support and coping ability.^8^ In fact, these factors predict HRQoL more than disease-related complications do.^9^ The presence of a chronic disease (including diabetes or hypertension) and its associated complications predicts poor general health and emotional well-being; yet social support predicts higher self-reported general and emotional health in these populations.^10^ In the Diabetes Study of Northern California (DISTANCE), higher levels of emotional support and social network were positively associated with disease-mediating factors, such as increased physical activity, diet, and foot examination.^11^ Other studies have confirmed that social health, emotional health, interpersonal relationships, coping, and mental health status impact on chronic disease management, HRQoL, and outcomes for adults with comorbid diabetes and hypertension.^8^,^12^ Furthermore, social integration—as well as interaction with friends, family, and others—predicts disease outcomes (including diabetes and hypertension) in the elderly.^13^

Yet despite knowledge of these relationships in the literature, limited studies have examined the association between mental health, social and emotional health, HRQoL, and disease outcomes (HbA1c and blood pressure) in adults with multimorbidity, specifically in Appalachia. Hence, this study examines the relationship between social/emotional factors (i.e., interpersonal relationships, emotional stress, and emotional health), HRQoL, and clinical factors (i.e., HbA1c and blood pressure) in adults with comorbid diabetes and hypertension in the only wholly Appalachian state, West Virginia.

## METHODS

### Study Design

The study utilized a cross-sectional design. Baseline data collected from a 12-week, community-based, waitlisted-randomized-control-trial diabetes and hypertension self-management program (DHSMP) were analyzed. The program was offered to adults with comorbid diabetes and hypertension.

### Diabetes and Hypertension Self-Management Program (DHSMP)

The DHSMP is an adaptation of the evidence-based curriculum of the American Association of Diabetes Educators (AADE-7) and the Joint National Committee (JNC) guidelines. The 12-week program consists of a 75-minute educational session every week led by experts and trained health coaches (HCs). Health coaches also provide weekly follow-up motivation, problem solving help, and general guidance to their designated participants on topics ranging from lifestyle management to diabetes/hypertension risks. Participants completed self-report surveys and assessments at baseline, 3 months, and 6 months after the intervention start date.

### Study Setting and Participants

The DHSMP was conducted at two church locations in Morgantown and Charleston, West Virginia, respectively. As part of a larger research project, this study used baseline assessments completed by 75 participants. The inclusion criteria were adults aged 18 years and older, with no physical activity limitations (for walking and stretching), and comorbid diabetes and hypertension. Pregnant or breastfeeding women and those with diagnosed mental illness, physical activity limitations, or contraindicated for the DASH diet were excluded.

### Data Procedures and Data Collection

Trained HCs and phlebotomists recorded participants’ height, weight, waist measurement, and blood pressure. Additionally, phlebotomists collected fasting blood in person at the intervention site (a church). Participants completed self-report surveys. A $25 gift card to a local grocery store was given as incentive for program evaluation/surveys with each assessment completion. All participants had a study code that was unique to them (a combination of their birth month, birthdate, first letter of their first name, and first letter of their middle name [or “x” if not applicable]) to maintain confidentiality of information in the dataset. The identifying code and participant names were stored in the principal investigator’s locked file cabinet.

### Measures

*Demographic factors* included age, gender, education level, income, and body mass index (BMI) calculated from participants’ height and weight.

*Health Related Quality of Life (HRQoL):* The 12-item Short-Form Health Survey (SF-12 v2), a validated measure of health status, measured overall HRQoL, which is composed of the physical component summary (PCS) and mental component summary (MCS).^14^,^15^ Participant responses were evaluated using eight HRQoL domains: general health (GH), physical functioning (PF), role (physical) functioning (RP), bodily pain (BP), vitality (VT), role (emotional) functioning (RE), mental health (MH), and social functioning (SF). This study utilized the RE and MH subscales to evaluate quality of life.

*Emotional Distress* as a result of diabetes was measured using the Diabetes Distress Screening scale (DDS).^16^ The 17-item DDS survey is a validated measure of diabetes distress, and participants rated their inconvenience on a six-point scale from *a very serious problem* (6) to *not a problem* (1). Overall DDS was measured as the sum of the 17 item scores and its four subscales: emotional burden (EB), physician-related distress (PD), regimen-related distress (RD), and interpersonal distress (ID). This study utilized the EB, RD, and ID subscales to evaluate diabetes distress.

*Self-rated mental health* data were collected from a single-item measure of self-rated mental health used as a population health measure. The item asked respondents to rate their mental health on a five-point scale from *excellent* (5) to *poor* (1).

*The clinical measures* included HbA1c and blood pressure. Systolic and diastolic blood pressure were categorized as *good control* or *poor control*, with 0 representing poor control (either or both systolic or diastolic blood pressure was equal to or above 130 and 80, respectively) and 1 representing good control (both systolic and diastolic blood pressure were below 130 and 80, respectively).

### Sample Size and Power Analysis

The a priori sample size calculation indicated enrolling 75 subjects with 10% dropout rate will allow clinically meaningful change in the primary outcome (HbA1c and blood pressure). For comparison between the 12-week DHSMP and wait-listed control group at 6-months post-intervention, a sample size of 75 participants would achieve 80% power to detect a 0.6-point difference in HbA1c and 2.0 mm/hg difference in blood pressure using a two-sided two-sample t-test at a significance level of 0.05. A convenience sample of 91 adults was screened for eligibility and enrolled in the program from 2018 to 2019. However, 16 participants did not complete the clinical assessment due to work-/family- /travel-related conflicts in the first 3 weeks, with a total sample size of 75.

### Statistical Analysis

Descriptive analysis was conducted on EB, RD, and ID of the diabetes distress scale (normalized to range from 0 to 100); RE; and MH of HRQoL. Self-reported mental health was categorized as fair/poor and good/very good/excellent. One-way ANOVA was utilized to examine differences in mental health categories as related to (1) demographic and clinical measures such as age, BMI, and HbA1c and (2) EB, RD, and ID subscales of DDS. Data were analyzed using SPSS 29.0. Statistical inferences were based on a significance level of *P* (two-sided) ≤ .05.

## RESULTS

### Demographic characteristics of participants

The sample was comprised of 75 participants with comorbid diabetes and hypertension. The mean age and BMI were 60.8 years ± 12.2 (range 23 to 85 years) and 36.4 ± 8.1 (range 24 to 58), respectively. Most of the participants were female (64.0%), had at least college-level education or higher (50.6%), were retired (42.7%), and reported annual family income less than $50,000 (62.2%) (Table 1). The average number of years lived with diabetes and hypertension was 12.2 (SD=10.4) and 17.9 (SD=12.9), respectively. In other words, participants’ average reported age of onset for diabetes was 48.5 years and age of onset for hypertension was 43.7 years.

### Clinical Measures

Table 1 shows the means and standard deviations for clinical measures of DHSMP participants, including HbA1c, systolic blood pressure, and diastolic blood pressure. The mean HbA1c was 7.4 ± 1.54 (range 5.1 to 12.2). Twenty six percent had poor glycemic control (HbA1c ≥ 8.0). The average blood pressure was 137.6/83.6 mmHg, with recorded systolic pressures ranging from 98 to 187 mmHg and diastolic pressures ranging from 64 to 124 mmHg.

Table 2 shows the mean age, BMI, HbA1c, and blood pressure of respondents by self-reported mental health categories (fair/poor and good/very good/excellent). One-way ANOVA was used to examine between-groups significance. Participants who self-reported fair/poor mental health had significantly higher BMI (41.8) than those who reported good/very good/excellent mental health (35.4; *F* = 7.6, *p = .008*). Table 2 also shows the mean DDS subscale scores for EB, ID, and RD (normalized to 100) by self-reported mental health (fair/poor and good/very good/excellent) using one-way ANOVA. While participants with fair/poor mental status had slightly higher diabetes-related distress scores than those who reported good to excellent mental health, the differences were not statistically significant (*P* > .05).

The HRQoL subscale scores for RE, MH, PCS, and MCS by mental health are also presented in Table 2. Results show that individuals with fair/poor mental health had significantly lower role/emotional function scores (RE) than those who reported good/very good/excellent mental health (*p <* .001). Furthermore, participants who self-reported fair/poor mental health also had significantly lower HRQoL mental health function scores (MH) at 38.3 than those who reported good/very good/excellent mental health at 48.2 (*F* = 10.8 (*p* = .002), *p* < .05).

For the physical component summary (PCS) score, participants who reported fair/poor mental health had significantly lower scores (36.5) than those who reported good/very good/excellent mental health (43.5) (*F* = 6.0 (*p* = .017), *p* < .05). The mental component summary (MCS) scores were also significantly lower for participants who reported fair/poor mental health compared to those who reported good/very good/excellent mental health—36.2 v. 49.1 (*F* = 19.5 (*p* = .000), *p* < .05).

## DISCUSSION

This is the first study among rural, Appalachian patients to characterize the relationship between self-reported mental health status, selected social and emotional health factors, and clinical biomarkers in adults with comorbid diabetes and hypertension. Findings confirm that participants with self-reported fair-to-poor mental health status have significantly lower quality of life than those with good-to-excellent mental health, especially in the domains of role (emotional) functioning, mental health, and overall HRQoL (physical and mental health). Findings concur with prior studies that show an independent relationship between mental health and chronic disease patients’ quality of life.^6^,^17^

To date, numerous studies have examined the association between various chronic conditions and HRQoL. However, most have focused on a particular group of patients or on certain chronic illnesses (e.g., cancer, heart disease, or diabetes) and the elderly in large urban areas.^9^,^18^–^21^ Very few studies have focused on rural Appalachian patients.^22^ The present study is also novel in its exploration of wide-ranging correlates of self-reported mental health. Limited studies have explored the correlates of self-reported mental health with BMI, HRQoL role/emotional functioning and overall mental health, or with clinical factors such as blood pressure.^19^,^20^,^23^

HRQoL and its domains are known, valid indicators. Lower HRQoL among patients with poor glycemic control—or high blood pressure in type 2 diabetes patients (when compared to controlled patients)—may be due to higher BMI, as obesity has been shown to decrease HRQoL in individuals with type 2 diabetes.^19^ Also, comorbid diabetes and hypertension have been shown to negatively impact all HRQoL dimensions.^23^ Obesity is specifically associated with lower MCS and PCS summary scores of HRQoL, and this association is strengthened for patients with comorbid hypertension.^24^ These findings indicate the potential interactions between obesity, mental health status, and HRQoL in patients with chronic conditions and support our results. BMI increases the risk for mental health issues and disorders. Additionally, morbid obesity was associated with emotional disorders among women in the general population.^24^,^25^

Findings also indicate that poor mental health status is inversely related to higher systolic blood pressure of participants (or conversely, participants with a lower blood pressure self-reported good mental health); this coincides with prior studies.^17^ While the question remains which particular chronic diseases have greater impact on HRQoL, participants in this study had multimorbidity (i.e., comorbid diabetes and hypertension), and 80% were obese (BMI ≥ 30). These results align with prior research among individuals in Central Appalachia that indicates poor mental health.^22^ In addition, a systematic review and meta-analysis authenticated that poor HRQoL is associated with mortality among individuals with chronic diseases.^18^ People living in Appalachia have multiple social determinants of health factors (e.g., lack of transportation, neighborhood safety and safe walking areas, and access to healthy food and food deserts) as well as patient level barriers (health insurance, poor self-efficacy and support) that impede compliance with clinical and self-care.^26^ More importantly, the associations between mental health and HRQoL were maintained even after adjusting for sociodemographic characteristics. Therefore, assessment of HRQoL and interventions is necessary at early stages of the chronic condition(s) to reduce disease-related complications.^6^ The results also reiterate the need for further mental health assessment, such as screening for depression and disease-related distress among rural patients, to mitigate negative impacts on HRQoL.

HRQoL in chronic patients focuses on three broad domains—physical, psychological, and social functioning—impacted by self-care and/or disease management and treatment.^6^ The rise in prevalence of chronic conditions and multimorbidity in West Virginia across the past decade, and the large number of people living with chronic diseases that are adversely affected by poor-to-fair quality of life, makes the findings only more relevant. Providers can use them to better plan and distribute healthcare resources, while incorporating patients’ perspectives to tailor HRQoL intervention approaches.

There are other lessons for primary care providers. Practitioners who are trained and practice within the biomedical paradigm of disease management tend to focus on physiological and clinical outcomes during clinical encounters (e.g., HbA1c and blood pressure).^6^ However, medical literature increasingly points to the benefits of promoting patient-centered communication, active listening, and relationship-building. Providers can work to be responsive to patient health concerns and attend to their beliefs and contextual factors.^27^ This way of working may be especially important for older patients with multimorbidity, and can foster bidirectional patient–provider communications for comprehensive treatment approaches and tailoring changes over time.^28^ These factors fall under the social science paradigm that includes HRQoL, overall functioning, and well-being. 6 Hence, provider trainings on collaborative care, care coordination, and measures that matter to patients—such as how to conduct HRQoL assessments and improve provider–patient communication—would be beneficial.

There have been encouraging, recent changes to clinical practice to bring collaborative care and the integration of mental health interventions, social support services, and primary care. This study’s findings—of the importance of positive mental and emotional health for self-management of chronic diabetes — support the importance of such integrated approaches with patients, especially those experiencing chronic disease and older patients with multimorbidity. In attending to these groups, it is important for healthcare providers to have bidirectional communications that enable comprehensive, adaptable treatment approaches.^28^ Future interventions may benefit from incorporating further knowledge of the relationship between mental health and multimorbidity management to improve patient knowledge and understanding and strengthen self-care and coping strategies. Ultimately, these can enable better health outcomes for those living with diabetes and hypertension, among other comorbidities.

### Limitations

The findings of this study are subject to several limitations. The generalizability is limited due to the small sample size and selection bias (i.e., educated and retired participants). The U.S. Census indicates that 23% of the population are college educated v. 50% in this study’s sample. In addition, 53% of the adults in West Virginia are in the labor force, compared to 43% of study participants. However, the population was comparable in household income, with 62.2% of study participants reporting less than $50,000 annually v. the West Virginia median household income of $55,217 for 2018–2022. Due to the cross-sectional nature of the study design, no causal inference could be established, and only associations among mental health, social or emotional health measures, and clinical measures (of HbA1c and blood pressure) were determined. Another limitation was the self-report survey design, which could introduce recall and social desirability biases. Additionally, the self-reported mental health distribution, for which participants were grouped into two categories (i.e., excellent/very good/good v. fair/poor), was not a normal distribution. Hence, participants who reported excellent/very good v. fair/poor may have very different characteristics within the same category.

Future studies should incorporate into their analyses additional factors (e.g., social support and social determinants of health) not explored in this study to provide a better understanding of mental health among adults with comorbid chronic conditions. Furthermore, longitudinal studies can provide information on whether self-management programs help patients cope with social/emotional factors related to disease management and thereby improve mental health. A larger study with a more heterogeneous and demographically representative patient population and additional assessments of factors that impact HRQoL could also be useful, in addition to mixed-methods analysis. Each of these enhancements could provide better understanding and guidance for tailoring future interventions specific to West Virginian adults with multimorbidity.

## IMPLICATIONS

The study aimed to identify the social/emotional factors affecting HRQoL and disease outcomes among community-based adults with multimorbidity in the Appalachian state of West Virginia. Findings show that obesity, especially morbid obesity, self-reported mental health, and high blood pressure were associated with poor HRQoL mental health indicators. These findings lay the groundwork for educational interventions, treatment, and self-care to be tailored to assess and address social/emotional and mental health among rural individuals with multimorbidity. In particular, the importance of positive mental and emotional health in diabetes self-management may be beneficial in sessions that focus on adherence to self-care and coping strategies in disease management planning. Future studies should replicate the program with a larger sample size to help confirm findings.

**SUMMARY BOX**

**What is already known about this topic?**

Diabetes and hypertension, two prevalent comorbid chronic conditions in West Virginia that require self-management for good health outcomes.^2^ Mental health plays a key role in the self-management of these patients. Diabetes and hypertension are linked to depression and anxiety disorders, conditions associated with compromised mental health status.^3^

**What is added by this report?**

The study aimed to identify the social/emotional factors affecting HRQoL and disease outcomes among community-based adults with multimorbidity in the Appalachian state of West Virginia. Findings show that obesity, especially morbid obesity, self-reported mental health as well as high blood pressure was associated with poor HRQoL mental health indicators.

**What are the implications for future research?**

The findings demonstrate a need for population level educational interventions, treatment, and self-care to address social/emotional and mental health among rural individuals with multimorbidity. Additional insights regarding positive mental and emotional health can help people with diabetes optimize adherence to self-care and coping strategies.

## Figures and Tables

**Table 1:** Clinical measures and demographics data for DHSMP participants (N = 75)

Clinical Measures		
	Mean	Standard Deviation
HbA1C	7.4	1.5
Systolic Blood Pressure	137.6	18.8
Diastolic Blood Pressure	83.6	10.9
**Demographics**		
	**Mean**	**Standard Deviation**
Age	60.8	12.2
BMI	36.4	8.1
Years With Diabetes	12.3	10.4
Years With Hypertension	18.0	12.9
	**Frequency**	**Percent**
**Gender**		
Male	27	36.0
Female	48	64.0
**Highest Education Level**		
High school graduate/GED	14	18.7
Some college/Technical school	15	20.0
Associate degree/Technical school graduate	8	10.7
College Graduate (BA, BS, BEd)	16	21.3
Graduate Degree (MA, MS, MD, PhD, DrPH)	22	29.3
**Employment**		
Working Full-time (35 or more hours/week)	28	37.3
Working Part-time (<35 hours/week)	4	5.3
Unemployed	3	4.0
Disabled/Unable to work	3	4.0
Homemaker	1	1.3
Retired	32	42.7
Self-employed	4	5.3
**Annual Salary**		
< $25,000	21	28.4
$25,000 $50,000	25	33.8
$50,000 $75,000	17	23.0
$75,000 $100,000	8	10.8
> $100,000	3	4.1

**Table 2:** Sample characteristics by self-rated mental health for DHSMP participants (N = 74)

	Poor/Fair (n = 13)		Good/Very Good/Excellent (n = 61)		Total (N = 74)			
	Mean	SD	Mean	SD	Mean	SD	*F*-value	*p-*value
**Demographics**								
Age	58.9	10.8	61.0	12.5	60.6	12.2	0.3	.590
BMI	41.8	9.8	35.4	7.2	36.5	8.1	7.6	.008 *
**Clinical Measures**								
HbA1c	7.7	1.7	7.3	1.5	7.4	1.5	0.6	.448
Systolic BP	143.7	19.1	135.9	18.6	137.3	18.8	1.9	.179
Diastolic BP	84.1	8.4	83.6	11.4	83.7	10.9	0.0	.884
**Diabetes Distress Screening (DDS)**								
Emotional Burden (EB)	32.6	35.7	18.4	21.4	20.9	24.8	3.7	.059
Regimen Distress (RD)	45.2	30.6	30.9	25.7	33.4	26.9	3.1	.081
Interpersonal Distress (ID)	19.0	28.5	14.2	22.6	15.1	23.6	0.4	.512
**SF-12 Health-Related Quality of Life (HRQoL)**								
Role/emotional Functioning (RE)	31.6	14.4	46.4	11.5	43.8	13.2	16.3	< .001*
Mental Health (MH)	38.3	12.3	48.2	9.3	46.4	10.5	10.8	.002*
Physical Component Summary (PCS)	36.5	12.2	43.5	8.7	42.2	9.7	6.0	.017*
Mental Component Summary (MCS)	36.2	10.2	49.1	9.5	46.9	10.7	19.5	< .001*

NOTE:

* Values are statistically significant at *p* = .05.
